# A Novel Open-Loop Tracking Strategy for Photovoltaic Systems

**DOI:** 10.1155/2013/205396

**Published:** 2013-11-12

**Authors:** Cătălin Alexandru

**Affiliations:** Transilvania University of Braşov, Boulevard Eroilor Nr. 29, 500036 Braşov, Romania

## Abstract

This paper approaches a dual-axis equatorial tracking system that is used to increase the photovoltaic efficiency by maximizing the degree of use of the solar radiation. The innovative aspect in the solar tracker design consists in considering the tracking mechanism as a perturbation for the DC motors. The goal is to control the DC motors, which are perturbed with the motor torques whose computation is based on the dynamic model of the mechanical structure on which external forces act. The daily and elevation angles of the PV module represent the input parameters in the mechanical device, while the outputs transmitted to the controller are the motor torques. The controller tuning is approached by a parametric optimization process, using design of experiments and response surface methodology techniques, in a multiple regression. The simulation and experimental results demonstrate the operational performance of the tracking system.

## 1. Introduction

The increase of the emissions of carbon dioxide, responsible for the global warming and for the greenhouse effect, may have devastating consequences on the environment. A solution is the renewable energy, with the solar energy conversion being one of the most addressed topics in the field. The photovoltaic (PV) systems convert solar energy into electric energy, with their efficiency depending on the degree of use and conversion of the solar radiation [1, 2]. Excepting the solar radiation, there are other parameters which have influence on the conversion efficiency of the PV cells, such as temperature and illumination.

The degree of use of the solar radiation can be maximized by use of tracking systems, which can increase the energetic efficiency up to 40–50% more than fixed systems [3–7]. The orientation principle of the PV modules is based on the input data referring to the position of the Sun on the sky dome. For the highest conversion efficiency, the sunrays have to fall normal on the receiver so the system must periodically modify its position in order to maintain this relation between the sunrays and the module. The positions of the Sun on its path along the year represent an input data in designing the solar trackers, so the geometrical relationship between the Earth and the Sun has to be considered. 

Consequently, for the design process of the tracking systems, two rotational motions can be considered, the daily motion and the yearly precession motion, resulting in two fundamental ways to track the Sun: by one axis (monoaxis) and by two axes (dual-axis). The monoaxis tracking systems spin on their axis to track the Sun, facing east in the morning and west in the afternoon; this type of tracker needs a seasonal tilt angle adjustment [8]. The dual-axis tracking systems follow the Sun more precisely due to the combination of the daily and seasonal/elevation motions; they are more efficient than the monoaxis systems but have the disadvantage of a higher price owed to their extra mechanical and electrical parts.

**Figure 1:**
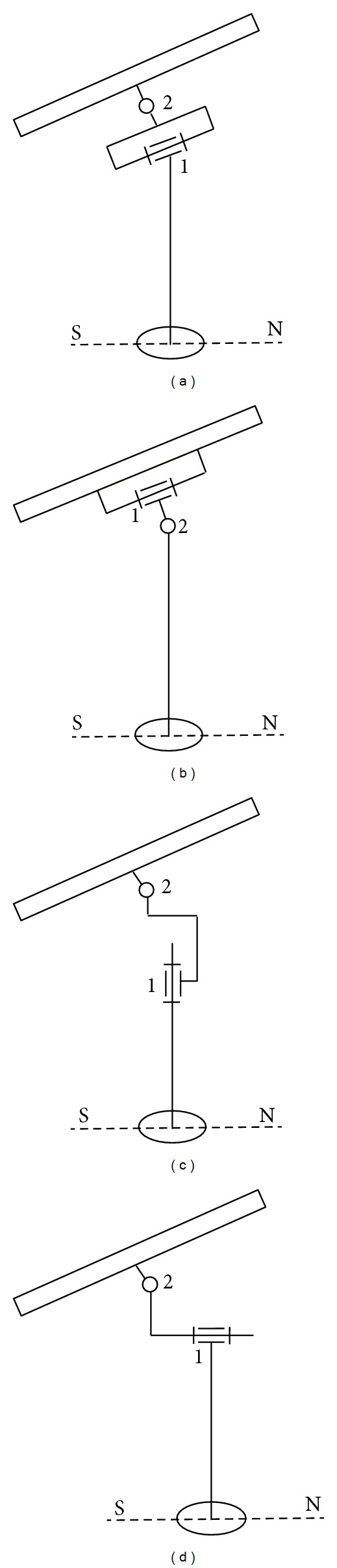
Dual-axis tracking systems.

Depending on the relative position of the revolute axes, four types of dual-axis systems can be identified (Figure 1, where “1” is daily motion, “2” is elevation, “S” is South, and “N” is North): equatorial (a), pseudo-equatorial (b), azimuthal (c), and pseudo-azimuthal (d). For the equatorial trackers, there are two independent motions, because the daily motion is made rotating the module around the polar axis. The pseudo-equatorial systems have an axis parallel with the revolute axis that determines the seasonal variation of the Sun position on the sky dome, with the revolute axis for the daily orientation oscillating relative to the seasonal axis. For the azimuthal trackers, the daily motion is made by rotating the module around the vertical axis, so that it is necessary to continuously combine the vertical rotation with an altitudinal motion around the horizontal axis. The pseudo-azimuthal tracker is derived from the azimuthal system, having the main/daily rotational axis positioned on the horizontal. From functional and constructive point of view, the equatorial and pseudo-equatorial systems represent the best solution to track the individual PV modules, while for the PV platforms and strings (or platforms of strings), azimuthal and pseudo-azimuthal systems are frequently used.

Many authors have adopted the open-loop control method as a basis in construction and design of tracking systems. The sun-tracking formula presented in [9] provides a general mathematical solution, improving the tracking accuracy by tackling the errors due to installation defects, which are analyzed from recorded solar images. Mehleri et al. [10] have developed a method to maximize the incident solar irradiance and to minimize the variance of the produced power as an optimization problem that aims to determine the optimum values of the orientation angles. The determination of the trajectories for an azimuthal system is approached in [11] as a nonlinear and bounded optimization problem, using a stochastic search algorithm (differential evolution). The method developed by Sungur [12] uses the azimuth and solar altitude angles for controlling an electromechanical system, which is controlled via a programmable logic control (PLC). Vişa et al. [13] have proposed a method to establish the open-loop orientation program for azimuthal systems, using minimal required number of changes in the azimuth and altitude angles.

In this paper, an equatorial tracking system (see Figure 1(a)) for individual PV module is approached. The study is focused on the control system design, the original aspect consisting in the consideration of the tracking mechanism as a perturbation for the driving motors, alongside with the disturbances coming from the external environment (e.g., the wind action), while in the previous researches from the literature only the external factors are used as perturbations. The goal is to control the driving actuators, which are perturbed with the motor torques whose computation is based on the dynamic model of the mechanical structure on which external forces act. The new control strategy has imposed an important modification on the modelling of the input and output plants. In the traditional control (including previous papers of the author [3, 14–16]), the input plants in the mechanical device model are the motor forces/torques (generated by the driving actuators) and the output plants (which are transmitted to the control system) refer to the position or velocity parameters (e.g., the daily position/angle of the PV module or the angular velocity of the rotor), while in the proposed control strategy the plants are just the opposite. Through this strategy, the perturbations can be handled much well, without the need to use complex (expensive) in-cascade control strategies.

The control system is an open-loop based on a mathematic algorithm that provides predefined parameters for the motors, depending on the Sun positions. These positions can be precisely determined because they are functions of the solar angles that can be calculated for any local area. By using this technique, the errors introduced by the use of the photosensors (for variable weather conditions), in the traditional closed-loop systems, may be avoided. 

## 2. Tracking System Design

For identifying accurate and efficient mechanical configurations suitable for tracking systems, a synthesis method based on the multibody systems (MBS) theory has been developed. A collection of possible structural schemes for tracking systems has been obtained, with the convenient solution being selected by a multicriteria analysis and with the evaluation criteria referring to the tracking accuracy, the motion amplitude, the complexity of the system, and the possibility for manufacturing. 

**Figure 2:**
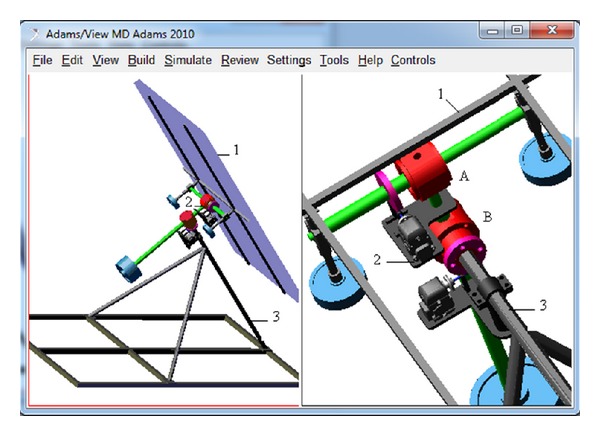
The MBS model of the equatorial tracking system.

The solution in study corresponds to a dual-axis equatorial system (Figure 2), whose revolute axis of the daily motion is fixed and parallel with the polar axis. There are two DC motors that drive speed reducer worm gears. The PV module “1” rotates (in the revolute joint A) around a support “2” for generating the seasonal/elevation motion. The support is connected to the fixed pillar “3” by a revolute joint (B), which allows the daily motion. The revolute joints materialize the bearings from the physical model. The DC motors are disposed by plates on the support (for the elevation motion), and, respectively, on the pillar (for the daily motion). The screw worm, whose shaft is coupled with the rotor, meshes with the worm wheel, which is rigidly connected to the module/support. The transmission ratio of the speed reducer is 50/1 for both motions. The direction of transmission is not reversible, due to the greater friction involved between the worm and worm wheel, by using a single start worm; this assures the self-locking in the stationary positions (between the motion steps).

The equilibration of the tracking mechanism has been made by using a system of counterweights, which are dimensioned and disposed in order to obtain the mass centres of the moving structures closed to the revolution axes. In this way, the motor torques for orienting the PV module in both directions are minimized, and this has a positive effect on the energy consumption for performing the tracking.

The solid model of the tracking mechanism, which contains information about the mass and inertia properties of the bodies, was realized using the CAD software SOLIDWORKS. For developing the dynamic model of the tracking mechanism, the solid model was transferred from the CAD environment to the MBS environment ADAMS (see Figure 2), which is used to analyze and simulate the mechanical device of the solar tracker by defining the constraints between the bodies and establishing the connection/communication with the control system model. The dynamic model of the tracking mechanism takes into consideration the mass forces, the joint reactions, and the joint frictions. The joint reactions are converted into equivalent torques using the friction arm and pin radius, while the joint bending moment is converted into an equivalent torque using pin radius divided by bending reaction arm.

The solar tracker is an automated (controlled) system, which has as task the orientation of the PV module (i.e., the effecter) on the imposed trajectory. For the tracking system in study, there are the following subsystems: the DC motors, the mechanical transmissions, and the mechanical structure. The connecting scheme of the subsystems emphasizes the following: the mechanism is a coupled system (each element has influence on the others); the transmissions are connected between the joints and the DC motors; the transmissions and the DC motors are uncoupled subsystems.

The design problem can be formulated in the following way: the control system must ensure the displacement of the effecter (i.e., the PV module) on the imposed trajectory, which can be transformed (through the use of inverse kinematics) in two imposed trajectories for the motor axes. Thus, the purpose is to design a control system that allows the rotations of the motor axes on the desired trajectories.

The mechanical transmission of the tracking system is modelled in the following way:

(1) $$\begin{array}{c} q_{m1/2}=i_{1/2}\cdot q_{1/2},\;\;T_{1/2}=\frac{1}{i_{1/2}}\cdot \tau_{1/2}, \end{array}$$

where *q*_1/2_ is angular position in the revolute joint, *q*_*m*1/2_ is angular position of the motor axis (rotor), *τ*_1/2_ is joint torque, *T*_1/2_ is motor torque, and *i*_1/2_ is gear transmission ratio.

The DC motor, which is an electromechanical system, is modelled by the following equations:

(2) $$\begin{array}{c} V=R_{a}i+L\frac{di}{dt}+E,\\ J{\ddot{q}}_{m}=K_{M}i-b{\dot{q}}_{m}-T,\\ E=K_{b}{\dot{q}}_{m}, \end{array}$$

where *V* is input voltage, *T* is torque, *R*_*a*_ is resistance, *L* is inductance, *i* is current, *K*_*b*_ is contraelectromotor constant, *q*_*m*_ is rotor position, ${\dot{q}}_{m}$ is rotor velocity, *J* is inertial moment, *K*_*M*_ is motor torque coefficient, and *b* is viscous friction. For this research, two identical DC motors (MAXON ROBOTIS DX) have been used for driving the daily and elevation motions of the PV module, with the following numerical values: *J* = 233*e* − 6 Nms^2^/rad, *L* = 0.5 H, *R*_*a*_ = 0.8 *Ω*, *b* = 0.1 Nms/rad, *K*_*M*_ = 176*e* − 3 Nm/A, and *K*_*b*_ = 0.105 Vs/rad.

**Figure 3:**
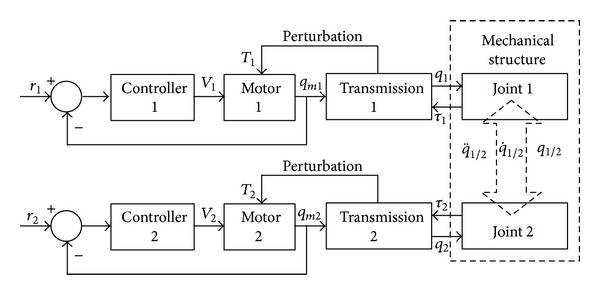
The basic block scheme of the tracking system.

The basic block scheme of the tracking system (Figure 3) emphasizes the connections between subsystems and the measures which define the communications. In accordance with this model, the goal is to control the DC motors (the reference measures of the control problem are the revolute angles of the motor axes *q*_*m*1/2_), which are perturbed with the torques *T*_1/2_ whose computation is based on the dynamic model of the mechanical structure on which outside forces act.

Equations (2) can be transformed with the Laplace operator (by eliminating the variable *I*(*s*)), as follows:

(3) $$\begin{array}{c} Q_{m}\left(s\right)=G_{V}\left(s\right)\cdot V\left(s\right)+G_{T}\left(s\right)\cdot T\left(s\right), \end{array}$$

where Ω_*m*_(*s*) is the Laplace transform of ${\dot{q}}_{m}$, *V*(*s*) is the Laplace transform of *V*, *T*(*s*) is the Laplace transform of *T*, while *G*_*V*_(*s*) and *G*_*T*_(*s*) are the transfer functions:

(4) $$\begin{array}{c} G_{V}\left(s\right)=\frac{K_{M}}{LJs^{3}+\left({JR}_{a}+bL\right)s^{2}+\left({bR}_{a}+K_{b}K_{M}\right)s},\\ G_{T}\left(s\right)=\frac{Ls+R_{a}}{LJs^{3}+\left({JR}_{a}+bL\right)s^{2}+\left({bR}_{a}+K_{b}K_{M}\right)s}. \end{array}$$

**Figure 4:**
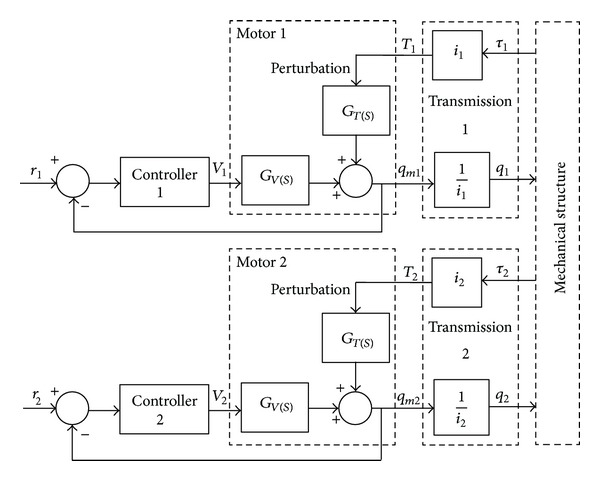
The refined control scheme of the tracking system.

The basic block scheme of the tracking system (shown in Figure 3) can be transformed into a more convenient scheme (Figure 4), the plant transfer function, in accordance with the adopted control strategy, being *G*_*V*_(*s*).

**Figure 5:**
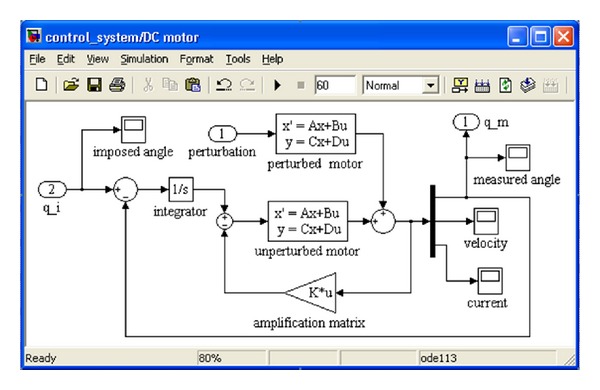
The block diagram of the DC motor.

The control strategy is based on the following statements: the tracking system is a complex system composed from three subsystems—DC motor, gear transmission, and mechanical structure; the DC motor is assimilated with the fixed part of the control problem, with the other subsystems being perturbation sources; a controller with a pole in origin is required to eliminate the perturbation. Under these circumstances, the block diagram model of the DC motor (which is designed in MATLAB/Simulink) is shown in Figure 5, where *q*_*i*_ is the imposed angular position (the reference signal for the daily/elevation angle), while *q*_*m*_ is the measured angle.

In the state space, the dynamic model of the DC motor can be expressed in the following form:

(5) $$\begin{array}{c} \dot{x}=\left[\begin{array}{ccc} 0 & 1 & 0\\ 0 & -\frac{b}{J} & \frac{K_{M}}{J}\\ 0 & -\frac{K_{b}}{L} & -\frac{R_{a}}{L} \end{array}\right]\cdot x+\left[\begin{array}{c} 0\\ 0\\ \frac{1}{L} \end{array}\right]\cdot u+\left[\begin{array}{c} 0\\ -\frac{1}{J}\\ 0 \end{array}\right]\cdot T,\\ y=\left[\begin{array}{ccc} 1 & 0 & 0 \end{array}\right]\cdot x, \end{array}$$

which can be segmented in two parts:

(i) the unperturbed part:
  (6) $$\begin{array}{c} \dot{x}=\left[\begin{array}{ccc} 0 & 1 & 0\\ 0 & -\frac{b}{J} & \frac{K_{M}}{J}\\ 0 & -\frac{K_{b}}{L} & -\frac{R_{a}}{L} \end{array}\right]\cdot x+\left[\begin{array}{c} 0\\ 0\\ \frac{1}{L} \end{array}\right]\cdot u,\\ y=\left[\begin{array}{ccc} 1 & 0 & 0 \end{array}\right]\cdot x, \end{array}$$
(ii) the perturbation effect:
  (7) $$\begin{array}{c} \dot{x}=\left[\begin{array}{ccc} 0 & 1 & 0\\ 0 & -\frac{b}{J} & \frac{K_{M}}{J}\\ 0 & -\frac{K_{b}}{L} & -\frac{R_{a}}{L} \end{array}\right]\cdot x+\left[\begin{array}{c} 0\\ -\frac{1}{J}\\ 0 \end{array}\right]\cdot T,\\ y=\left[\begin{array}{ccc} 1 & 0 & 0 \end{array}\right]\cdot x. \end{array}$$

The following state vector is used for the controller modelling:

(8) $$\begin{array}{c} x=\left[\begin{array}{ccc} q & \dot{q} & i \end{array}\right], \end{array}$$

where *q* is angular position, $\dot{q}$ is angular velocity, and *i* is current (intensity). The controller tuning, intending to determine the optimal values of the amplification matrix terms, will be approached in the next section of the paper.

On the next stage, the DC motor model (developed in MATLAB/Simulink; see Figure 5) and the MBS mechanical device model (developed in ADAMS; see Figure 2) have been integrated at the virtual prototype level (in mechatronic concept). For connecting the mechanical device model and the control system model, the input and output plants have been defined. The daily and elevation angles of the PV module represent the input parameters in the mechanical model, while the outputs transmitted to the controller are the torques that perturb the DC motors. The daily angle is measured in the revolute joint between the intermediary support and the fixed pillar, while the elevation angle is measured in the revolute joint between the PV module frame and the intermediary support (see Figure 2).

For the input state variables, the run-time functions are 0.0 during the simulation, because the variables will get their values from the control application. The run-time functions for these variables are VARVAL (daily angle) and VARVAL (elevation angle), where VARVAL is a specific ADAMS function that returns the value of the given variable; in other words, the input daily and elevation angles get the values from the input variables. For the output state variables, the run-time functions return the sum of torques at locations.

**Figure 6:**
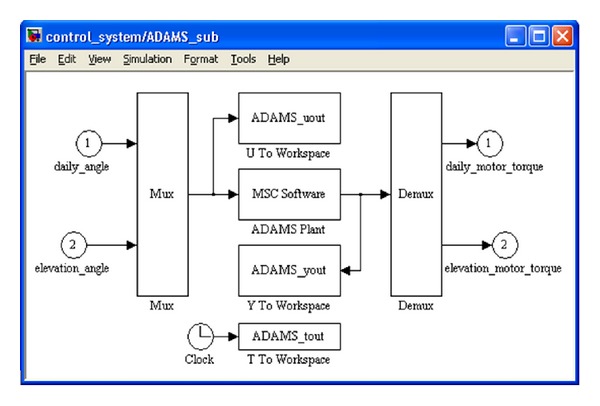
The mechanical device block in MATLAB/Simulink.

Then, the ADAMS plant files have been exported for the control application using the ADAMS/Controls interface. The Plant Inputs refer to the input state variables (the daily and elevation angles), and the Plant Outputs refer to the output state variables (the motor torques). ADAMS/Controls save the input and output information in a specific file for MATLAB (.*m*); this file is used to create the ADAMS_sub block (Figure 6), which contains the S-Function representing the nonlinear ADAMS model (i.e., the MBS mechanical device).

**Figure 7:**
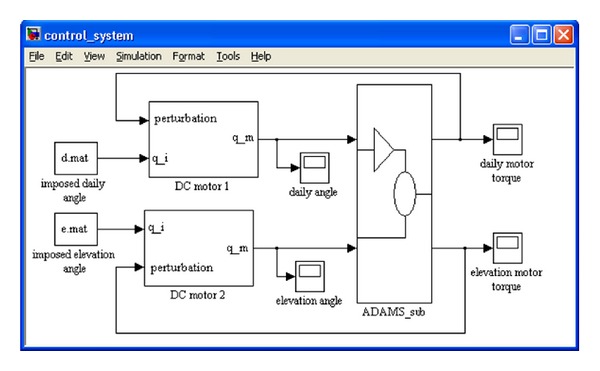
The block diagram of the control system.

Finally, the control system model (Figure 7) is obtained by integrating the ADAMS_sub block, the DC motors blocks, and the input functions blocks (containing the desired trajectory of the PV module). In the mechatronic model, ADAMS accepts the control inputs from MATLAB and integrates the mechanical model in response to them. At the same time, ADAMS provides the motor torques for MATLAB to integrate the control system model.

## 3. Optimal Design of the Controller

The literature presents several methods for the controller design (in order to obtain the optimum controller parameters), such as Ackermann algorithm [17], Pareto solution [18], or PSO [19]. In this paper, the optimal design of the controller is performed in a parametrical optimization process based on DOE (design of experiments) and regression models, by using ADAMS/Insight. The optimization purpose is to minimize the tracking errors (the difference between the imposed daily/elevation angle *q*_*i*_ and the measured angle *q*_*m*_; see Figure 5), with the monitored value being the root mean square during simulation. 

The data transfer from MATLAB to ADAMS is realized by generating an external system library-ESL (.dll) from the control system. Once imported in ADAMS as a general state equation (GSE), the parameterized model of the control system (Figure 7), coupled with the MBS model of the tracking system (Figure 2), becomes available for optimization, in the form of an experiment type file (.xml) for ADAMS/Insight. 

The optimization has been performed in the following stages: modeling the design objective/response (the root mean square of the tracking errors during the simulation—RMS_error); modeling the design variables/factors (the amplification matrix [*K*_1_, *K*_2_, *K*_3_]); setting the investigation strategy and planning a set of trials in which the factor values vary from one trial to another; executing the runs and recording the performance of the system at each run; fitting the results to a response surface by a multiple regression; evaluating the results soundness (i.e., goodness of fit); optimizing the system, which involves reaching the minimum values of the response. For each factor, the variation field (minimum and maximum values) in the optimization process has been defined as follows: *K*_1,2,3_ ∈ [1, 200].

For the tracking system in study, several DOE investigation strategies (screening, response surface) and design types (full factorial, Plackett Burman, d-optimal) have been tested, with the aim to identify the factors and combinations of factors that most affect the response. The best results (in terms of goodness of fit) have been obtained for the DOE response surface strategy with full-factorial design. This method fits polynomials to the results of the trials in the experiment. Full factorial is the most comprehensive of the design types and uses all of the possible combinations of factor levels. The total number of runs is *m* ^*n*^, where *m* is the number of levels and *n* is the number of factors. In this case, considering the minimum and maximum values of the three factors, an experiment with 8 runs (trials) has been obtained.

Based on the design specifications, the design space and the work space have been created. The design space (Table 1) is a matrix with the rows representing the runs and the columns representing the factors settings, which are in a normalized representation (e.g., “−1” corresponds to the minimum value of the factor, while “1” is for the maximum value). The work space (Table 2) is a matrix with the rows indicating the runs and the columns identifying the factors settings and resulting response values.

The paper presents the exemplification for the daily motion. The imposed angle was modeled as a time function, with the PV module rotating 10 degrees in 60 seconds (*d*.*mat* block in Figure 7). For each trial, ADAMS performs a simulation, with the results appearing in the work space (see Table 2).

Then, the work space is used to establish a relation between factors and response, by fitting the results to a response surface. The relationship between factors and response is modeled by a response surface (linear, quadratic or cubic), the best results (with regard to goodness of fit) being obtained for the linear model with interactions, whose effects are captured through special terms in the model that consist of products of factors. The corresponding regression model is defined by the following equation:

(9) RMSerror=a0+a1·K1+a2·K2+a3·K3 +a4·K1·K2+a5·K1·K3+a6·K2·K3,

where *a*_0_, *a*_1_, …, *a*_6_ are the coefficients calculated by multiple regression analysis (with *a*_0_ being the coefficient of the constant term).

The goodness of fit is defined by the following statistical measures [20]: *R*-squared (*R* ^2^), *R*-squared-adjusted (*R*_adj_^2^), regression significance (*P*), range-to-variance (*R*/*V*), and *F*-ratio (*F*). *R*-squared indicates the variance in the predicted results versus the real data. This is the proportion of total variability in the data which is explained by the regression model, with a score of “1” indicating a perfect fit. *R*-squared-adjusted is similar to *R*-squared but is adjusted to account for the number of terms. Regression significance indicates the probability that the fitted model has no useful terms, with small values (less than “0.01”) indicating that the fit does have useful terms. Range-to-variance ratio indicates how well the model predicts values at the data points. *F*-ratio is used in the regression to test the significance of the regression, with high values suggesting that the regression is significant and the model is useful.

**Figure 8:**
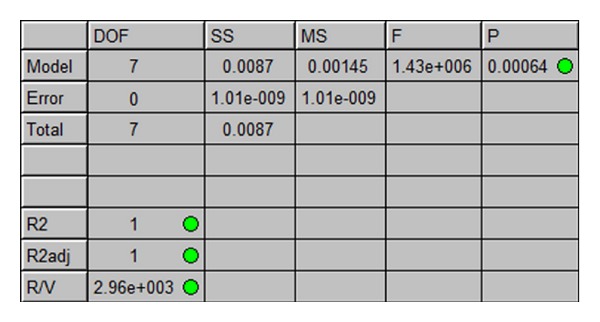
The fit table for the regression model.

The results shown in Figure 8 indicate that the regression model for the selected strategy (DOE response surface, full-factorial, linear with interactions) matches the test data very well, with the “green” bullets indicating the results soundness (entitiy is likely appropriate). The fit table contains also the number of independent variables that goes into the estimation of a parameter (DOF), the sum of squares (SS), and the mean square (MS), for the three parts of the statistical model—regression (model), residual (error), and total.

**Figure 9:**
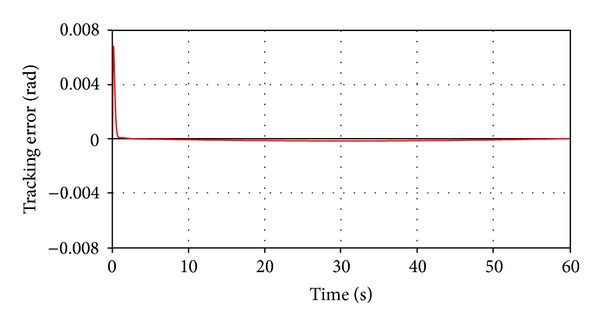
The tracking error during simulation.

The method used in optimization is OptDes-GRG (generalized reduced gradient), which is a conventional gradient-based optimizer. During optimization, the factors are adjusted so that the resulting response (the root mean square of the tracking error) comes as closely as possible to the specified target value. Using the operator LsEq (less than or equal) with the target value 10^−3^, the controller amplification matrix will result in a simulation that meets the design requirements, *K* = [5.9, 106.6, 187.4], with the time-history variation of the tracking error being shown in Figure 9.

**Figure 10:**
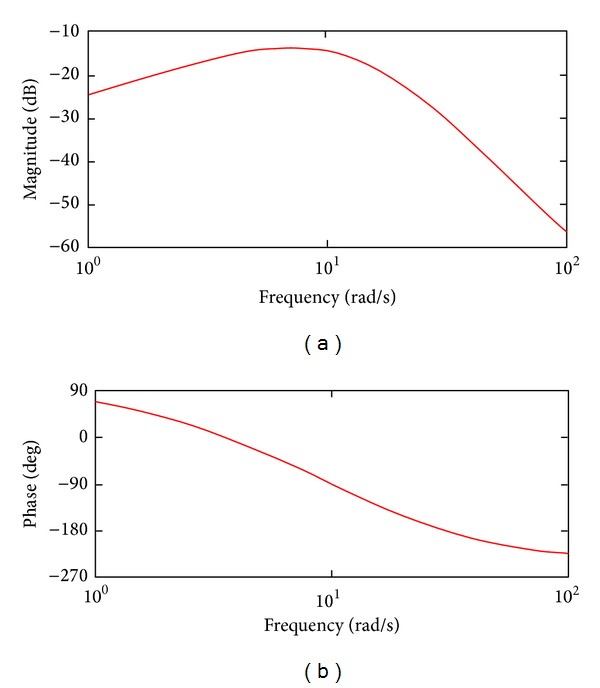
The frequency response of the system.

The performance of the proposed tracking system is also proved by the frequency response, which is the measure of the system's output spectrum in response to the input signal (the magnitude of the system response and the phase versus frequency). This was obtained by plotting the magnitude and phase measurements through the Bode plot (Figure 10). The system works like a filter, with the amplification being less than 3 dB. The natural frequency of the controlled system is *ω*_*n*_ = 7.66 rad/sec, corresponding with the amplification of −13.8 dB; consequently, the system filters out very well. 

## 4. Evaluating the Energetic Efficiency of the Tracking System

The main task in the design process of the solar trackers is to maximize the energetic gain by increasing the solar input and minimizing the energy consumption for tracking. The PV module can be rotated without brakes during the daylight or can be moved in steps (step-by-step). The maximum incident radiation can be obtained for the continuous motion, but in this case the operating time of the motor is high and there are necessary large transmission ratios; another disadvantage is the behaviour of the system in terms of occurrence of external perturbations, such as the wind or snow action. On the other hand, the direct solar radiation has small values in the limit positions, closed by the sunrise and sunset, and for this reason it is not efficient to track the Sun in these areas. In these conditions, a step-by-step tracking strategy was developed considering the correlation between the optimal motion field and the number of the steps. The idea is to minimize the angular field and the number of steps without significantly affecting the incoming solar radiation.

The solar radiation is influenced by parameters specific to the geographical area, such as season, hour, climatic conditions, degree of pollution, and turbidity factor (atmosphere clarity). The incident radiation, which is normal to the active surface, is given by the following relation:

(10) $$\begin{array}{c} R_{I}=R_{D}\cdot \cos\alpha , \end{array}$$

where *R*_*D*_ is the direct terrestrial radiation and *α* is the angle of incidence. 

The mathematical model for estimating the direct solar radiation, which is based on the Meliß's empirical approach [21], was presented and validated in [15]. The angle of incidence was determined from the scalar product of the sunray vector and the normal vector on the PV module,

(11) $$\begin{array}{c} \alpha =\cos^{-1}\left(\cos\beta \cdot \cos\beta^{*}\cdot \cos\left(\gamma -\gamma^{*}\right)+\sin\beta \cdot \sin\beta^{*}\right), \end{array}$$

in which *β* and *γ* are the diurnal and seasonal angles of the sunray, while β* and γ* are the daily and elevation angles of the PV module. The quantity of energy produced by the PV module, with (*E*_*T*_) and without (*E*_*F*_) tracking, depends on the quantity of incident solar radiation (*R*_*I*_), the active surface (*S*), and the conversion yield (*η*) of the module,

(12) $$\begin{array}{c} E_{T/F}=S\cdot \eta \cdot \int_{t_{0}}^{t}R_{I}\;dt. \end{array}$$

The strategy for the tracking law design intends to identify the optimal angular motion field of the PV module, the number of motion steps, and the actuating timing. The solution is obtained by implementing an optimal algorithm in ADAMS, based on the parametric design technique. The imposed tracking law (which gives the daily angle variation) was modeled in ADAMS as a sum of STEP functions. STEP is a run-time function, with the following format:

(13) $$\begin{array}{c} \text{STEP}\left(x,x_{0},f_{0},x_{1},f_{1}\right), \end{array}$$

where *x* is the independent variable (time), *x*_0_ is the value of independent variable at which the function begins, *f*_0_ is the initial value of the step, *x*_1_ is the value of independent variable at which the function ends, and *f*_1_ is the final value of the step (relative to the initial value). 

Because the solar radiation curves are symmetricaly relative to the noon, the motion law will be also symmetrical (with positive values of the daily angle in the morning and negative values in the afternoon). Due to this consideration, a noticeable facilitation is introduced in the motion law design, considering just half of law (from sunrise to noon). Then, the motion law is transposed for the tracking from noon to sunset by inverting the daily angle sign.

Thus, the following daily motion half law (from sunrise to noon) was modeled:

(14) STEP(time,(tr+t1),β0∗,(tr+t1+Δt),−Δβ∗)  +STEP(time,(tr+t1+t2),0,(tr+t1+t2+Δt),−Δβ∗)+⋯  +STEP(time,(tr+t1+t2+⋯+tn),0,(tr+t1+t2+⋯+tn+Δt),−Δβ∗),

where *n* is the number of steps for the half law, *t*_*r*_ is the sunrise time, *t*_1_ is the time at which the first motion step begins, relative to *t*_*r*_, *t*_2_ is the time at which the second motion step begins, relative to the first step (*t*_*r*_ + *t*_1_), *t*_*n*_ is the time at which the final step begins, relative to the preceding step, Δ*t* is the motion step duration (for simplification, there was considered the same duration for all steps, Δ*t* = 60 seconds), *β*_0_* is the initial value of the daily angle, Δβ* is the step size (for simplification, all the motion steps have the same size, Δβ* = *β*_0_*/*n*). 

In the optimization process, the following design variables have been defined: the initial value of the daily angle (*β*_0_*), the number of steps for the half law (*n*), and the relative actuating times (*t*_1_, *t*_2_,…, *t*_*n*_). The design objective is a numerical representation of the energetic efficiency of the PV system with sun tracker (*ε*), which is expressed in the following way:

(15) $$\begin{array}{c} \varepsilon =E_{T}-\left(E_{F}+E_{C}\right), \end{array}$$

where *E*_*T*_ is the energy produced by the PV system with tracking, *E*_*F*_ is the energy produced by the fixed (nontracked) equivalent system, and *E*_*C*_ is the energy consumption for tracking. The optimization purpose is to maximize the design objective (energetic efficiency) value at simulation end.

The algorithm used in optimization is similar to that used in the previous section for the optimal design of the controller (based on DOE investigation strategy and multiple regression analysis), and it will be detailed in a future paper. As a result of the optimization process, the optimal values of the design variables (for the half law, from sunrise to noon) have been obtained. Then, the half law is transposed for the daylight tracking (the whole law, from sunrise to sunset). For this paper, the numerical simulations have been performed for the summer solstice day, considering the Braşov geographic area (location latitude is 45.5°, declination angle is 23.45°, sunrise local time is 5.466, sunset local time is 21.183, and noon local time is 13.206). 

**Figure 11:**
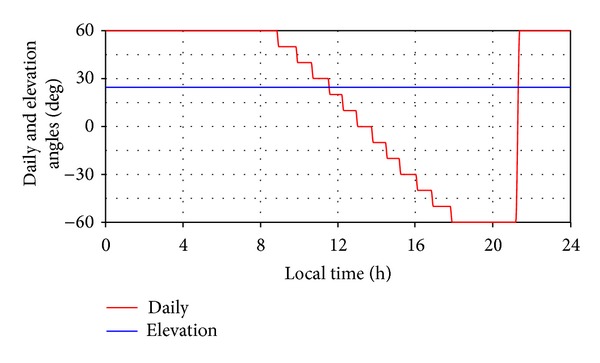
The motion laws of the PV module (daily and elevation angles).

Under these circumstances, the tracking law for the daily motion is defined in the following way (Figure 11): the angular field—β* ∈ [60°, − 60°]; 12 tracking steps (for the whole law), meaning Δβ* = 10° for each step; actuating timing (in local time)—8.839, 9.819, 10.619, 11.489, 12.189, 12.919, 13.719, 14.449, 15.149, 16.019, 16.819, and  17.799. The return in the initial position (sunrise) is made at sunset (*t*_*s*_ = 21.183), with continuous motion. The seasonal (elevation) angle is fixed at γ* = 24.5° (relative to the horizontal position of the PV module).

**Figure 12:**
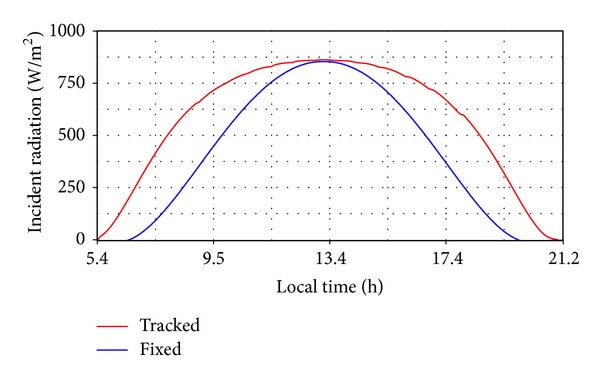
The incident radiation curves (with and without tracking).

**Figure 13:**
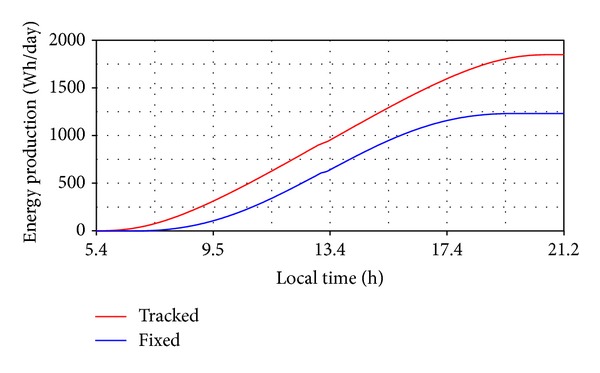
The energy production (with and without tracking).

Considering these values and the mathematical model for estimating the solar radiation, the incident radiation curves have been obtained (Figure 12), for the PV system with tracking (a), as well as for the PV system without tracking (b), which is fixed in the noon position (β* = 0°,  γ* = 24.5°). Afterwards, the energy production was determined for a PV module with the active surface *S* = 1.26 m^2^ and the conversion yield *η* = 15% as follows (Figure 13): *E*_*T*_ ≈ 1848 Wh/day and *E*_*F*_ ≈ 1230 Wh/day.

The energy consumption for realizing the tracking was obtained through the dynamic simulation of the virtual prototype of the mechatronic tracking system (see Section 2), depending on the measured voltage (*u*) and current (*i*),

(16) $$\begin{array}{c} E_{C}=\int_{t_{0}}^{t}u\cdot i\;dt. \end{array}$$

**Figure 14:**
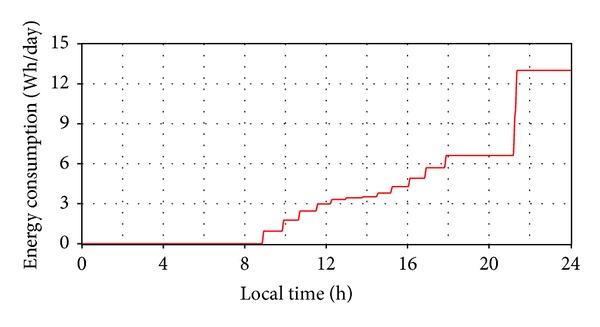
The energy consumption for the daily motion.

The time-history variation of the energy consumption is shown in Figure 14, resulting in *E*_*C*_ ≈ 13 Wh/day (only for the daily motion, with the seasonal/elevation position being fixed). In this way, the energetic efficiency of the system is obtained, *ε* = *E*_*T*_ − (*E*_*F*_ + *E*_*C*_) = 1848 − (1230 + 13) = 605 Wh/day, representing an energetic gain of 49.2% relative to the fixed PV module. Similar computations have been performed for different days/periods throughout the year, with the average annual gain being around 35%. This value demonstrates the viability/utility of the tracking system in increasing the energetic efficiency of the PV module.

## 5. Testing the Physical Prototype

**Figure 15:**
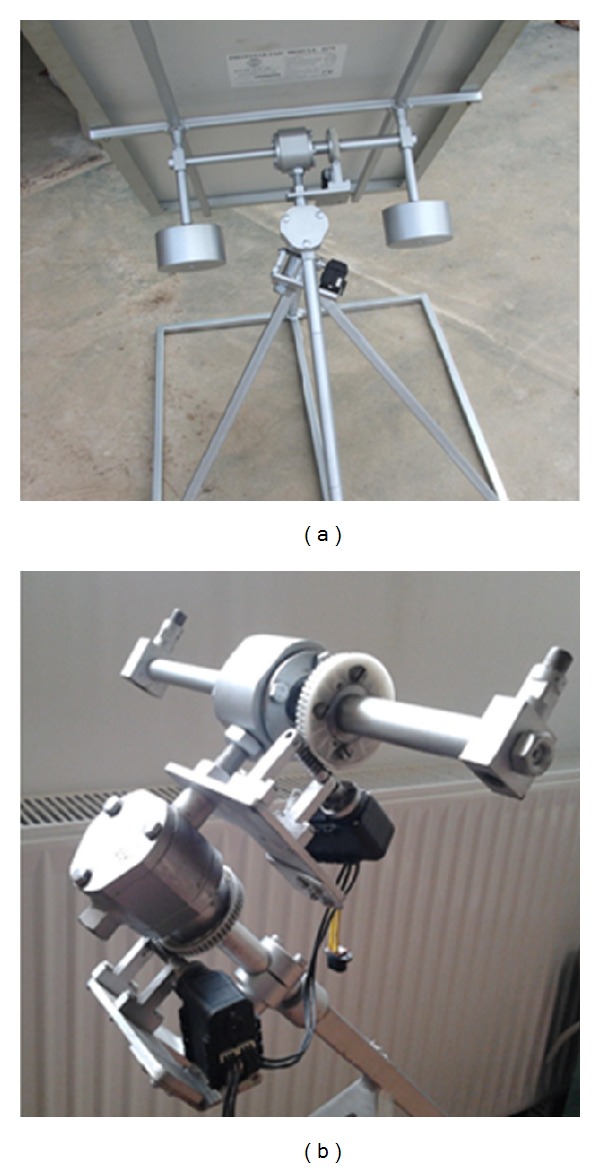
The physical prototype of the equatorial tracking system.

The modelling and simulation in virtual environment precede the manufacturing of the physical prototype (experimental model). The physical prototype of the equatorial tracking system (Figure 15) has been manufactured, and it will be installed in the Green Energy Independent University Campus (GENIUS) from the Transilvania University of Braşov. MAXON ROBOTIS DX-117 motors are used to drive the system (for the daily and elevation motions), in accordance with the proposed open-loop tracking strategy, which was implemented by using the NI LabVIEW platform (the detailed implementation will be presented in a future paper). 

In this section of the paper, the experimental model is used to evaluate the robustness performance of the tracking system (i.e., the capability to operate with the imposed indexes, or close to these values, when one or more parameters of the physical model are changing). The goal is to verify the behaviour of the system in terms of occurrence of nonstationary external perturbations, such as wind or heavy snow load. Wind action is usually evaluated either by wind pressure or by wind forces. Wind effects depend on wind properties, shape, dimensions, and position of PV systems towards direction of wind [22, 23]. 

For this study, a wind gust with the speed of 30 m/s (the value is representative for Braşov area—from the measurements with the local weather station)—has been considered; this corresponds to a pressure of 1.73 kN/m^2^. For the PV module in study, with the area *S* = 1.26 m^2^ (1.5 × 0.84 m), a wind force of 2.18 kN is obtained. 

**Figure 16:**
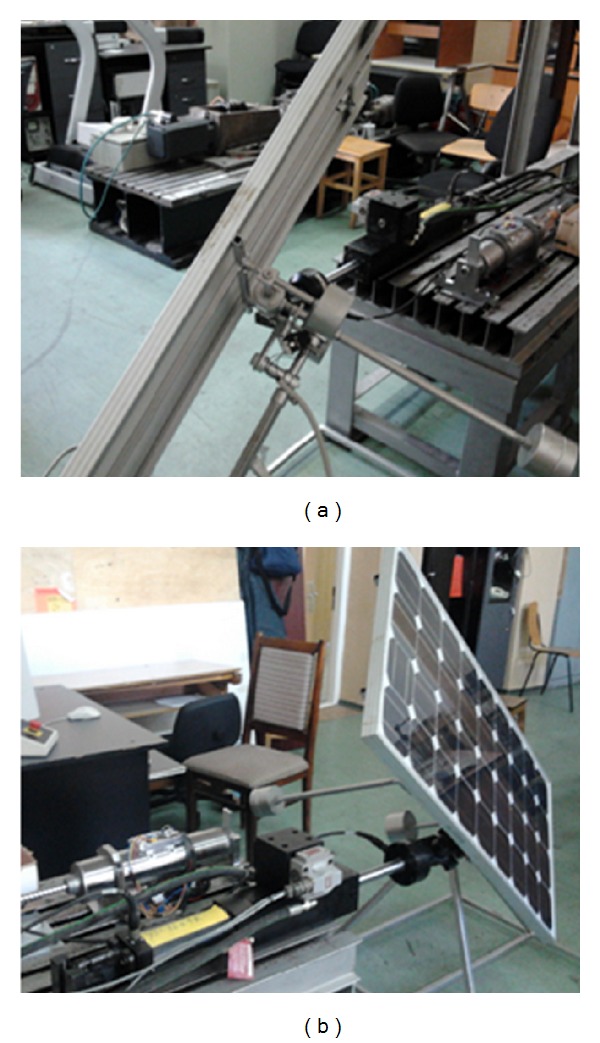
The experimental test for simulating the wind action.

For the current research stage, the response of the tracking system to wind action is tested in laboratory conditions. The wind force has been “generated” with a hydraulic linear actuator (part of the MTS/HTC hydraulic test equipment from the Product Design, Mechatronics and Environment Department). The hydraulic actuator is able to generate a force of ±25 kN, with the maximum stroke being ±125 mm (with control in force or in displacement). The actuator piston is connected to PV module frame (Figure 16), applying a force that simulates the wind action.

**Figure 17:**
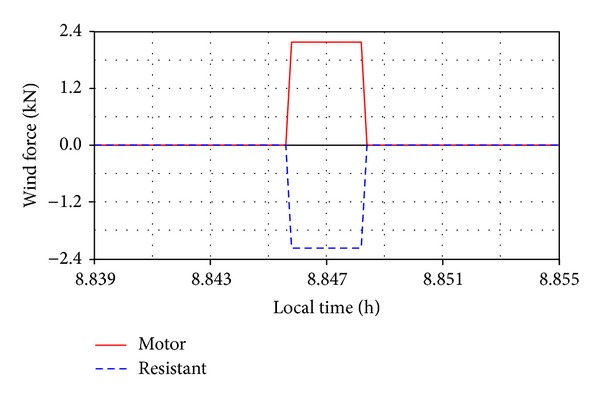
The time-history variation of the applied force: (a)—motor, (b)—resistant.

In the above-mentioned wind conditions, a trapezoidal force profile has been considered, with the time-history variation being shown in Figure 17. The force is applied when the PV module is moving, on a portion of 10 seconds during the first daily motion step, β* ∈ [60°, 50°] (see Figure 11). The sign depends on the force direction (from the front or back relative to the PV module, meaning forward or backward displacement of the hydraulic actuator). The applied force generates a torque on the revolute axis, which can be in the same direction with the PV module motion (a) or in opposite direction (b).

**Figure 18:**
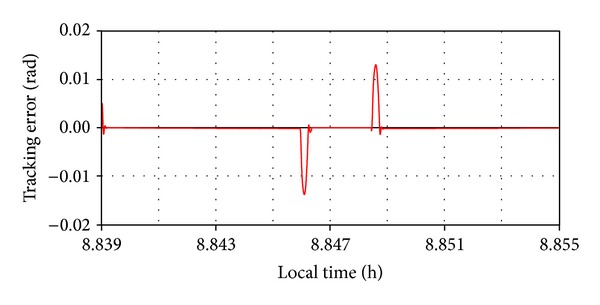
The tracking error for motor force.

**Figure 19:**
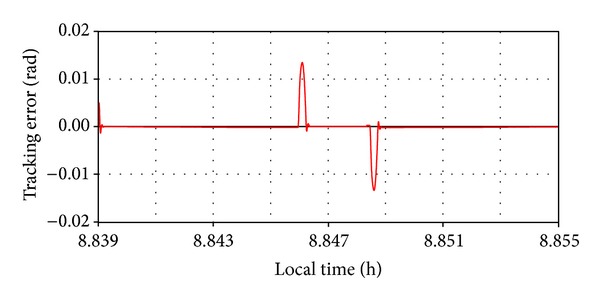
The tracking error for resistant force.

For both situations (depending on the applied force direction), the tracking error (the difference between the imposed and current daily angles) was used to evaluate the robustness performance of the tracking system, with the results being presented in the diagrams shown in Figures 18 and 19. Small errors (in acceptable limits) can be observed in the initial and final stages of the force action; otherwise, the system will settle down very well. The stationary error, after performing the motion step, is extremely low (practically zero). All these demonstrate a very good stability and robustness performance of the tracking system.

## 6. Conclusions

The proposed tracking/control strategy has three important features: generality, simplicity, and performance. The results demonstrate the performance of the tracking system (in terms of precision, stability, and robustness), without the need to use complex/expensive in-cascade strategies. Besides the equatorial tracking systems (such as that approached in the paper), the strategy can be applied/adapted for any other type of tracking mechanism (pseudo-equatorial, azimuthal, or pseudo-azimuthal). 

The simulations prove the importance of the virtual prototyping in the design process of the tracking systems, having as the main advantage the possibility of performing virtual measurements for any parameter, in any point or area. Another significant advantage brought by the virtual prototyping is the simplicity of the procedures with a reduced testing time and small cost relative to the traditional physical prototyping-based methods. 

The future researches will add more climatic parameters to the simulations (considering the diffuse solar radiation and variable atmospheric conditions). At the same time, the modelling of the mechanical structure with finite elements will be approached, for identifying the eigenshapes and eigenfrequencies of the system (which are useful to avoid the resonance phenomenon due to the action of the external dynamic loads). The energetic efficiency of the photovoltaic system will be evaluated by considering experimental data, and in this way a relevant comparison between the virtual prototype analysis and the data achieved by measurements will be possible.

## Figures and Tables

**Table 1:** The design space of the experiment.

Trial	*K*_1_	K_2_	K_3_
1	−1	−1	−1
2	−1	−1	1
3	−1	1	−1
4	−1	1	1
5	1	−1	−1
6	1	−1	1
7	1	1	−1
8	1	1	1

**Table 2:** The work space of the experiment.

Trial	*K*_1_	*K*_2_	*K*_3_	RMS_error [rad]
1	1	1	1	0.07034
2	1	1	200	0.01245
3	1	200	1	0.08212
4	1	200	200	0.02431
5	200	1	1	0.1003
6	200	1	200	0.09584
7	200	200	1	0.1007
8	200	200	200	0.09623

## References

[1] Chinnaiyan VK, Jerome J, Karpagam J (2013). An experimental investigation on a multilevel inverter for solar energy applications. *Electrical Power and Energy Systems*.

[2] Singh GK (2013). Solar power generation by PV (photovoltaic) technology: a review. *Energy*.

[3] Alexandru C (2013). Design and optimization of a mono-axial tracking system for photovoltaic modules. *Journal of Solar Energy*.

[4] Jeevandoss CR, Kumaravel M, Kumar VJ A novel method for the measurement of the C-V characteristic of a solar photovoltaic cell.

[5] Rahman S, Ferdaus RA, Mannan MA, Mohammed MA (2013). Design & implementation of a dual axis solar tracking system. *American Academic & Scholarly Research Journal*.

[6] Seme S, Štumberger G, Voršič J (2011). Maximum efficiency trajectories of a two-axis sun tracking system determined considering tracking system consumption. *IEEE Transactions on Power Electronics*.

[7] Yamin AH, Ibrahim MN, Idroas M, Zin AR Embedded solar tracking instrumentation system.

[8] Calabrò E (2013). An algorithm to determine the optimum tilt angle of a solar panel from global horizontal solar radiation. *Journal of Renewable Energy*.

[9] Chong KK, Wong CW (2009). General formula for on-axis sun-tracking system and its application in improving tracking accuracy of solar collector. *Solar Energy*.

[10] Mehleri ED, Zervas PL, Sarimveis H, Palyvos JA, Markatos NC (2010). Determination of the optimal tilt angle and orientation for solar photovoltaic arrays. *Renewable Energy*.

[11] Seme S, Štumberger G (2011). A novel prediction algorithm for solar angles using solar radiation and Differential Evolution for dual-axis sun tracking purposes. *Solar Energy*.

[12] Sungur C (2009). Multi-axes sun-tracking system with PLC control for photovoltaic panels in Turkey. *Renewable Energy*.

[13] Vişa I, Diaconescu DV, Duţă A, Popa V PV tracking data needed in the optimal design of the azimuthal tracker’s control program.

[14] Alexandru C, Pozna C (2010). Simulation of a dual-axis solar tracker for improving the performance of a photovoltaic panel. *Proceedings of the Institution of Mechanical Engineers, Part A*.

[15] Alexandru C, Tatu IN (2013). Optimal design of the solar tracker used for a photovoltaic string. *Journal of Renewable and Sustainable Energy*.

[16] Ioniţă MA, Alexandru C (2012). Dynamic optimization of the tracking system for a pseudo-azimuthal photovoltaic platform. *Journal of Renewable and Sustainable Energy*.

[17] Ackermann JE (1977). On the synthesis of linear control systems with specified characteristics. *Automatica*.

[18] Gao Q, Chen J, Wang L, Xu S, Hou YL (2013). Multiobjective optimization design of a fractional order PID controller for a gun control system. *The Scientific World Journal*.

[19] Yau HT, Lin C-J, Liang Q-C (2013). PSO based PI controller design for a solar charger system. *The Scientific World Journal*.

[20] Gonzáez-Manteiga W, Péez-Gonzáez A (2006). Goodness-of-fit tests for linear regression models with missing response data. *Canadian Journal of Statistics*.

[21] Meliß M (1997). *Regenerative Energiequellen: Praktikum*.

[22] Velicu R, Lateş M, Moldovean G Loading cases and forces on azimuthal solar tracking systems with linear actuators.

